# Metabolite Characteristics Analysis of Siliques and Effects of Lights on the Accumulation of Glucosinolates in Siliques of Rapeseed

**DOI:** 10.3389/fpls.2022.817419

**Published:** 2022-02-16

**Authors:** Farah Kamal, Shulin Shen, Ran Hu, Qianwei Zhang, Nengwen Yin, Yifang Ma, Yuxiang Jiang, Xinfu Xu, Jiana Li, Kun Lu, Cunmin Qu

**Affiliations:** ^1^Chongqing Engineering Research Center for Rapeseed, College of Agronomy and Biotechnology, Southwest University, Chongqing, China; ^2^Academy of Agricultural Sciences, Southwest University, Chongqing, China; ^3^Engineering Research Center of South Upland Agriculture, Ministry of Education, Chongqing, China

**Keywords:** *Brassica napus* L., siliques, metabolic analysis, shading treatment, glucosinolates

## Abstract

Glucosinolates (GSLs) are naturally occurring secondary metabolites found in the *Brassicaceae* family, which mainly synthesize in the siliques with a wide range of functions. In this study, we investigated the effects of lights on metabolites in siliques of rapeseed through ultra high-performance liquid chromatography (UPLC)—heated electrospray ionization (HESI)–tandem mass spectrometry (MS/MS). A total of 249 metabolites, including 29 phenolic acids, 38 flavonoids, 22 GSLs, 93 uncalculated and 67 unknown compounds, were identified in siliques of rapeseed. Meanwhile, 62 metabolites showed significant differences after shading treatment, which were mainly GSLs and unknown compounds. Interestingly, the amounts of 10 GSLs had high accumulation levels in siliques, while the expression levels of their corresponding biosynthetic genes (*AOP*, *GSL-OH*, *IGMT*, and *ST5a*) were obviously reduced after shading treatment. Further evidence showed that the amounts of GSLs were significantly reduced in seeds, in accordance with the expression profiles of transporter genes (*BnaGTRs*). Our findings indicated that lights could affect the accumulation and transportation of GSLs from siliques to seeds in rapeseed. Therefore, this study facilitates a better understanding of metabolic characteristics of siliques and provides insight into the importance of light for GSLs accumulation and transportation in siliques and seeds of rapeseed.

## Introduction

*Brassica napus* known as an important oilseed crop is cultivated worldwide, which can be used as a source of vegetable oil and protein meal (Shahid et al., 2019), as well as the production of biofuel, lubricant for steam engines and in the cosmetic industry (Qu et al., 2013; Yin et al., 2019). Meanwhile, rapeseed is also famous due to the presence of numerous secondary metabolites, and several hydroxycinnamic acid derivatives had been identified in the seeds, especially sinapic derivatives that are predominantly phenolic compounds in *Brassica* species (Cartea et al., 2010; Khattab et al., 2010; Szydłowska et al., 2010; Park et al., 2019). In addition, these compounds had been given much attention by scientists because of their health-promoting effects (Vaughn, 1999; Fahey et al., 2001). Among them, glucosinolates (GSLs) are sulfur-containing bioactive compounds usually present in *Brassica* vegetables (Park et al., 2019), which could also produce a pungent flavor and reduce their nutritional values (Justen, 2010; Qu et al., 2020).

On the basis of amino acids, their types, and structures, GSLs are usually characterized into three classes, such as aromatic GSL, aliphatic GSL, and indolic GSL (Ishida et al., 2014). The core structure of all these GSLs is comprised of β-thioglucose linked with (Z)-*N*-hydroximinosulfate ester through a sulfur atom and amino acid-derived side chains. These side chains with variable length and modifications determine the chemical properties of all the GSLs (Bell et al., 2018). To date, almost 200 different GSLs have been reported in plants and these GSLs rich plants mostly belong to *Brassicaceae* family (Clarke, 2010; Ishida et al., 2014). These endogenous metabolites play important roles in plant defense against different biotic (fungi, bacteria, nematodes, and insects) and abiotic factors (wounding, temperature, chemical deficiencies, UV radiations, and extreme light) (Chhajed et al., 2020). In addition, isothiocyanates, the breakdown products of GSLs in *Brassica* act as anticarcinogenic and prevent cardiovascular diseases in humans (Zang et al., 2009; Wang et al., 2011). Moreover, they also interact with other crucial metabolic pathways such as the auxin pathway and control growth and development (Nintemann et al., 2017). Therefore, it is necessary to attain a good balance of GSLs accumulation in *Brassica* species.

In plants, the pathway of GSLs has been studied extensively (Halkier and Gershenzon, 2006; Nguyen et al., 2020), which mainly involves three steps: chain elongation, core structure formation, and secondary modification (Lu et al., 2018; Chhajed et al., 2020) with some common genes, enzymes, and transcriptional factors (Sønderby et al., 2010; Chhajed et al., 2020). Therefore, the pathway of GSLs has been known as a “model” for secondary metabolites in *Arabidopsis* (Sønderby et al., 2010), including 52 *GSL* genes, providing the important clues for explaining the profiles and accumulation of GSLs in *Brassica* species (Wang et al., 2011). For example, previous studies showed a high colinearity in the GSL biosynthetic pathway between *Arabidopsis thaliana* and *Brassica rapa* (Zang et al., 2009; Wang et al., 2011); similarly, alkenyl hydroxyalkyl producing-2 (*GSL-ALK*), and methylthioalkylmalate synthase (*MAM1*, *MAM2*, and *MAM3*), were involved in different types of GSLs biosynthesis in *Brassica oleracea* and *Brassica napus* (*B. napus*) (Li and Quiros, 2002; Liu et al., 2010), respectively. In addition, the contents of GSLs showed significant differences among *Brassica* vegetables (Assefa et al., 2019), which were further varied depending on the cultivar genotypes (Kushad et al., 1999; Lee et al., 2014), growing conditions (Du and Halkier, 1998; Rosa and Rodrigues, 1998; Volden et al., 2008) and developmental stages (Brown et al., 2003). For example, the timing, intensity, and duration of light treatment could be used as a parameter to measure the GSLs fluctuations during different developmental stages (Kirkegaard et al., 2018). In *B*. *napus*, silique walls are the dominant photosynthetic organ for supporting the seed filling during late stages (Tan et al., 2020), which were acted as one of the main parameters for yield determination. Previous studies showed that the siliques are also the main production sites for GSLs biosynthesis in rapeseed and which are later transported from silique walls (source) to the seeds (sink) (Du and Halkier, 1998; Jørgensen et al., 2015). However, how light induces GSLs synthesis and transport in silique walls and seeds remains unclear.

To analyze the role of light for the synthesis and transport of GSLs, especially the GSLs metabolites and genes involved in photoregulation, we investigated the accumulation of GSLs metabolites and the expression levels of GSLs genes in rapeseed silique walls under normal and completely shading treatments. The results showed that light plays important role in the accumulation of GSLs in silique walls and seeds. Furthermore, the profiles of related genes under shading and normal conditions were investigated using RNA sequencing (RNA-seq) analysis and validated through reverse transcription-quantitative PCR (RT-qPCR) analysis, which showed highly consistent results with the accumulation patterns of GSLs in silique walls and seeds. This study provided the first detailed comparison of GSLs metabolites in silique walls and seeds under normal and shading conditions in rapeseed, confirming that the GSLs production was affected by light, which obviously inhibited the accumulation and translocation of GSLs from silique walls to seeds. Our findings provide clues for elucidating the GSLs biosynthesis and lay a foundation for improving the quality of rapeseed.

## Materials and Methods

### Plant Growth and Sample Collection

The rapeseed cultivars, GH06 and ZY821, with higher GSLs contents (>130 μmol g^–1^), were grown under normal field experimental conditions in Beibei (106.38°E, 29.84°N), Chongqing, China. Each accession was grown in randomized complete blocks with three rows at each site (0.4 m between rows and 0.2 m between plants). To investigate the dynamic metabolites of the silique walls during maturation, the flowers were marked with different color wools to keep the siliques at the same development stages. After 15 days of pollination (15 DAP), the siliques were completely covered with aluminum foil (Shading treatment), and the corresponding sides were uncovered as the control. After 20 days (35 DAP) of shading treatment, siliques were sampled and kept on ice before the removal of seeds. The fresh siliques and seeds were immediately kept in liquid nitrogen and then stored at −80°C until the extraction of RNA and metabolites, respectively.

### Chemical Standards and Calibration Curve

HPLC graded with formic acid, methanol and acetonitrile were used for HPLC–heated electrospray ionization (HESI)–tandem mass spectrometry (MS/MS) analysis. The commercial standards, such as sinigrin, sinapic acid, quercetin, *p*-coumeric acid, isorhamnetin, kaempferol, epicatechin, ferulic acid, and caffeic acid were at least liquid chromatography/mass spectrometry (LC/MS) grade (purity > 99%, Sigma-Aldrich Trading Corporation, Ltd., Shanghai, China). The stock solutions of standards were prepared individually in 80% methanol and stored in the dark at −20°C. A mixed stock standard solution of standards was then prepared in 80% methanol at 5 mg l^–1^ with respect to each standard. Spiked calibration curves at eight levels (0.001, 0.005, 0.01, 0.05, 0.20, 0.50, 1.0, and 2.0 mg l^–1^) were prepared in triplicate for calibration curve construction (Yin et al., 2019; Qu et al., 2020).

### Metabolites Extraction From Siliques

The raw metabolites extraction was performed as in our previously described studies (Yin et al., 2019; Qu et al., 2020) with minor modifications. In brief, fresh siliques stored at −80°C were weighed about 100 mg and crushed into powder using a high-throughput tissue grinder (Tissuelyser-192, Shanghai, China). Then, 1 ml extracting solution (80% aqueous methanol with 0.1% formic acid) was added to each sample and homogenized by vortex for 10 s. The mixed samples were extracted by KQ sonication (Kunshan, China) at 4°C for 1 h. The raw extractions were followed by centrifugation at 8,000 *g* at 4°C for 30 min and the supernatant was preserved at −80°C. Subsequently, the above process was repeated again under the same conditions. The two supernatants were pooled and filtered with 0.22 μm nylon filter for HPLC-HESI-MS/MS analysis.

### Ultra High-Performance Liquid Chromatography–HESI–MS/MS Analysis

The metabolite analysis was conducted as previously described by Yin et al. (2019) and Qu et al. (2020). Briefly, the Dionex UltiMate 3000 UHPLC system (Thermo Fisher Scientific, Waltham, MA, United States) coupled to a Thermo Scientific Q-Exactive System equipped with an S-Lens ionizer source (Thermo Fisher Scientific, Waltham, MA, United States) was used to analyze the extracted metabolites in negative mode. The metabolites were separated by an Acquity ultra high-performance liquid chromatography (UPLC) BEH C18 column (150 mm × 2.1 mm × 1.7 mm, Waters, Ireland) with a guard column (1.7 μm particle size, 2.1 mm × 5 mm, Waters, Ireland), thermostated at 40°C. The mobile phase is solution A (0.1% formic acid in water, v/v) (A) and B (0.1% formic acid in acetonitrile, v/v). The mobile-phase gradient was 0–2 min, 5–10% solution B; 2–10 min, 10–25% solution B; 10–13 min, 25–95% solution B; 13–16 min, 95% solution B; 16–16.5 min, 95–5% solution B; and 16.5–21 min, 5% solution B. The flow rate was set to 0.300 ml/min and the injection volume was 10 μl. Mass spectrometry was determined by the negative model with the full MS-ddMS^2^ method ranging from 100 to 1,500 (m/z). The gas of sheath, auxiliary, and sweep was set to 35, 10, and 0, respectively. The source voltage was 3.5 kV with 350°C capillary temperature.

### Data Processing and Metabolites Identification

Raw UPLC–HESI–MS/MS data were converted for free MS data analysis by ABF (Analysis Base File) converter^1^ and then analyzed using MS-DIAL version 4.18 software with mass bank negative database^2^ (Tsugawa et al., 2019). In addition, raw UPLC–HESI–MS/MS data were confirmed by Xcalibur 3.1 software based on retention times (RTs), accurate MS, and MS/MS spectral data, together with the commercial standards and previously reported information. Meanwhile, these unequivocally identified compounds were quantified using external calibration curves of respective chemical standards, and the unknown compounds were quantified using base peak area.

### Transcriptome Sequence Analysis

Total RNA was extracted from 100 mg of polled siliques of five individual plants using an EZ-10 DNAaway RNA Mini-Preps Kit (Sangon Biotech Corporation, Ltd., Shanghai, China). The quantity and quality of the purified RNA were examined with a Nanodrop spectrophotometer (Thermo Fisher Scientific, Waltham, MA, United States) and an Agilent 2100 Bioanalyzer (Agilent Technologies Incorporation, Santa Clara, CA, United States), respectively. The qualified RNA from each sample was then used for library construction according to the standard protocols and sequenced using an Illumina HiSeq2000 sequencing platform (Tianjin Novogene Bioinformatic Technology Corporation, Ltd., Tianjin, China). Two biological replicates were used for the transcriptome analysis. The RNA-seq data have been deposited to the National Center for Biotechnology Information (BioProject ID PRJNA770894).

After removing adapters and unknown or low-quality bases, clean reads were mapped to the *B*. *napus* cultivar “ZY821” genome (unpublished) using hierarchical indexing for spliced alignment of transcripts (HISATs) (version 2.1.0) (Kim et al., 2015) and the number of mapped reads was quantified using HTseq (high-throughput sequencing data) (Anders et al., 2015). The gene expression profiles were evaluated using fragments per kilobase of exon model per million (FPKM) values. Genes were considered as differentially expressed genes (DEGs) with a minimum twofold difference in expression [| log2 fold change (FC)| ≥ 1]. The heatmap of gene expression levels was generated using HemI 1.0 (Heatmap Illustrator, version 1.0) software (Deng et al., 2014).

### Reverse Transcription-Quantitative PCR Validation

Total RNA was isolated from the same samples that were used for metabolites extraction. First-strand cDNA was synthesized by an RNA PCR Kit (AMV, v3.0) based on the standard protocols of the manufacturer (Takara, Dalian, China). In this study, *AOP*, *GSL-OH*, *IGMT*, and *ST5a* from GSLs biosynthetic pathways and *BnaGTRs* genes for GSLs transport (Supplementary Table 4) were selected and validated by RT-qPCR. The RT-qPCR analysis was performed on the Bio-Rad CFX96 Real-Time System (Bio-Rad Laboratories, Hercules, CA, United States) using the SYBR qPCR SuperMix Plus (Novoprotein, Beijing, China) and the detailed procedures were as previously described (Qu et al., 2013). The *B*. *napus BnACTIN7* (*EV116054*) was used as an internal control gene for calculating the relative expression levels *via* the 2^–ΔΔCt^ method (Wu et al., 2010). All experiments were detected with biological triplicates and the values were represented by mean ± SD. The specific primers for the selected genes were designed from two different locations and the internal control gene (*BnACTIN7*) are given in Supplementary Table 5.

### Statistical Analysis

Each experiment for this study was performed with two independent biological replicates for a reproducible study. The univariate statistical analysis like principal component analysis (PCA) was carried out by using metaboanalyst^3^. In the univariate analysis, metabolite screening was performed using *q*-values and FC values. Then, accumulation pattern of these differential metabolites was represented by volcano plots of both the samples under normal and shading through GraphPad Prism version 8.0.1 software (GraphPad Software, San Diego, CA, United States). The metabolite annotation was carried out through the Kyoto Encyclopedia of Genes and Genomes (KEGG) for GSL pathway selection. All the data are represented using mean ± SD values of two replicative values.

## Results

### Characterization of Siliques Under Normal and Darkness

**FIGURE 1:**
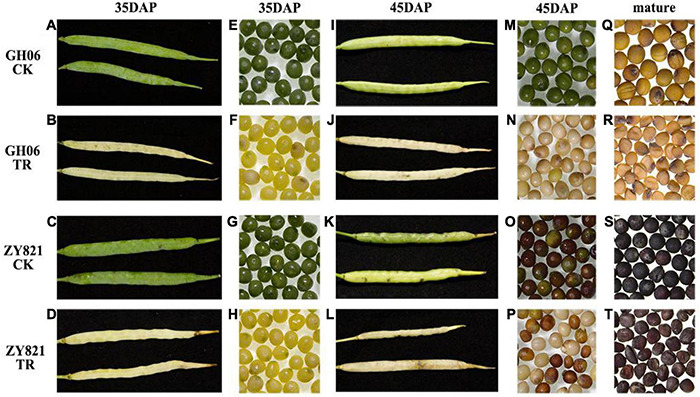
Phenotypic analysis of the siliques and seeds of rapeseed under different conditions. Siliques of GH06 and ZY821 under control **(A,C)** and shading **(B,D)** at 35DAP. Seeds of GH06 and ZY821 under control **(E,G)** and shading **(F,H)** at 35DAP. Siliques of GH06 and ZY821 under control **(I,K)** and shading **(J,L)** at 45DAP. Seeds of GH06 and ZY821 under control **(M,O)** and shading **(N,P)** at 45DAP. Mature seeds of GH06 and ZY821 after control **(Q,S)** and shading treatment **(R,T)**. CK, the control; TR, shading treatment; DAP, days after pollination. Scale bars = 1 cm.

Previous studies showed that light is essential for rapeseed seed development, and severe light deficiency could result in shrunken seeds by decreasing their storage reserves (Tan et al., 2015). In order to understand the importance of light for metabolites changes in siliques of rapeseed, we examined the phenotypic changes of GH06 and ZY821 under the control and shading treatment. Results showed that shading treatment clearly affected the color change in developing siliques and seeds (Figure 1). Apparently, the phenotypes of siliques and seeds showed the similar effects between the GH06 and ZY821, which lost the green color under shading as compared to control at 35DAP (Figures 1A–H). At 45DAP, phenotypes of shaded siliques remained the same, and the seeds of shading treated plants showed premature senescence, color differences, and size reduction (Figures 1I–P). Meanwhile, the mature seeds harvested after shading and control are shown in Figures 1Q,T. Obviously, the mature seeds of GH06 and ZY821 under treatment were light yellow and brown in color and wrinkled in shape as compared to healthy, round-shaped dark yellow and black seeds of control, respectively. Our findings indicate that light is an essential factor for the morphological development and biosynthesis of storage reserves in seeds, consistent with similar observations in *B*. *napus* (Tan et al., 2015, 2020).

### Metabolite Profiling in Siliques of *Brassica napus*

Previously results showed that siliques are the dominant photosynthetic organ for supporting the seed filling during late stages (Tan et al., 2020). To elucidate the effects of light on the biosynthesis of storage reserves of siliques for contributing to seed development of *B*. *napus*, we analyzed the accumulation of metabolites in siliques under the control and shading treatment by UPLC–HESI–MS/MS with negative mode. PCA demonstrated the reliability and reproducibility among the biological replicates of the same sample types by the UPLC–HESI–MS/MS (Supplementary Figure 1). First, a total of 3,245 base peak chromatograms were identified from siliques of GH06 and ZY821 using MS-DIAL version 4.18 software (Supplementary Figure 2). In this study, we identified 249 discernible peaks on the basis of their RTs, exacting MS, MS^2^ spectral data, and available standards (Supplementary Table 1). These distinguished peaks of metabolites were generally classified into six groups, including the phenolic acids, flavonoids, GSLs, lipids, hydroxycinnamic acids, and unknown compounds.

**FIGURE 2:**
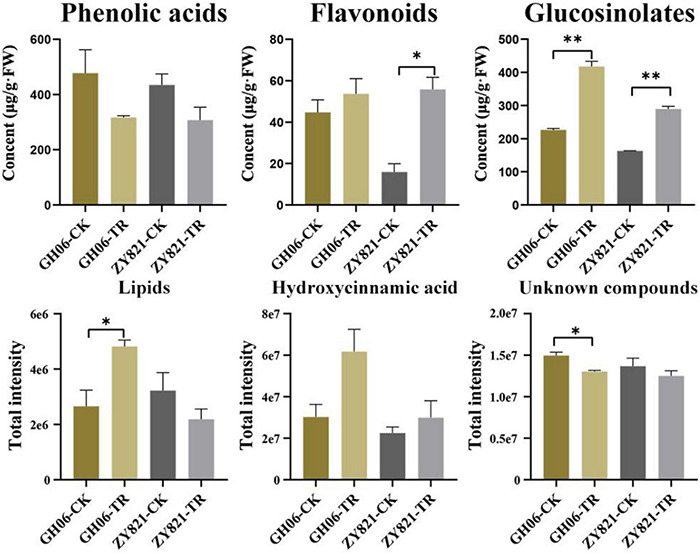
The total contents of detected compounds in rapeseed siliques under the control and shading treatment at 35DAP. Error bars represent the means ± SD, and the asterisks represent significant differences from the control, with *P < 0.05 or ^**^P < 0.01. CK, the control; TR, shading treatment.

Based on their mass spectrum, the concentrations of identified compounds were quantified using the calibration curves obtained from their corresponding or similar standards in this study (Supplementary Table 2). Results showed that the concentrations of total flavonoids, GSLs, and hydroxycinnamic acid increased in siliques under shading treatment than the control (Figure 2), of which GSLs were increased by 86.34% in GH06 and 57.89% in ZY821 compared with that of CK (Figure 2), respectively. Our findings suggested that the composition or transportation of metabolites in siliques could be affected by the light.

### Identification of the Differential Metabolites in Siliques of *Brassica napus*

**FIGURE 3:**
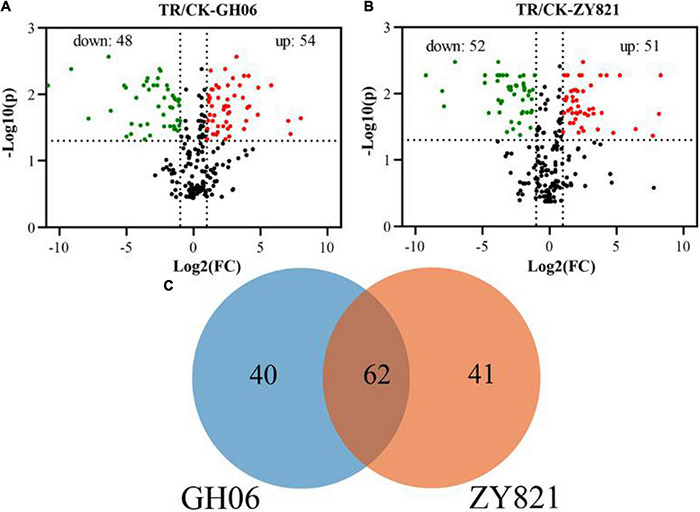
The differential metabolites in siliques of rapeseed. **(A,B)** Volcano plots of differential metabolites in siliques of GH06 and ZY821 under shading treatment and the control, each point in a volcano plot represents a metabolite; the abscissa represents the logarithm of the quantitative difference multiples of a metabolite in two samples; and the ordinate represents the variable importance in project (VIP) value, CK, the control; TR, shading treatment. **(C)** Venn diagram of differential metabolites in siliques of GH06 and ZY821.

In this study, the differential metabolites were screened from GH06 and ZY821 with a *P*-value ≤ 0.05, and Log2(Fold Change) ≥ 1 for each group. Herein, a total of 102 significantly differential metabolites in GH06 (48 downregulated, 54 upregulated) (Figure 3A and Supplementary Table 3) and 103 in ZY821 (52 downregulated, 51 upregulated) (Figure 3B and Supplementary Table 3). These metabolites were considered to be representative of differential characteristics between the shading treatment and normal light conditions. Among these metabolites, we further detected 62 common differential metabolites in both groups and unique compounds of GH06 (40) and ZY821 (41) (Figure 3C), including 3 phenolic acids, 3 flavonoids, 12 GSLs, 3 hydroxycinnamic acids, 7 lipids, and 34 unknown compounds, respectively (Supplementary Table 3). Moreover, these results provide the insight into well understanding the mechanisms of the metabolite accumulation involved in normal light conditions. As such, further investigation is needed to explore these differential metabolites in the future.

### Shading the Siliques Reduced the Transportation of Glucosinolates From Siliques to Seeds in *Brassica napus*

**FIGURE 4:**
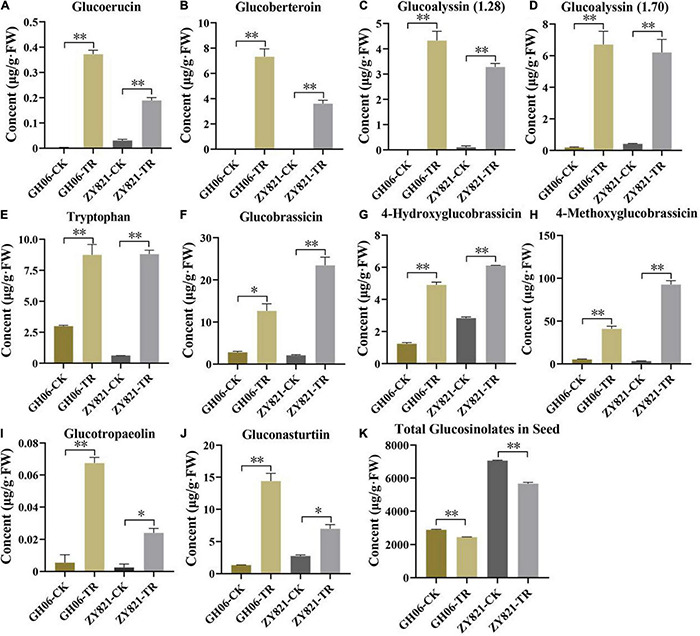
The GSLs profiles with significant differences in rapeseed siliques. **(A–J)** The contents of 10 GSLs in siliques of GH06 and ZY821 at 35DAP. **(K)** Total contents of GSLs in seeds of GH06 and ZY821 at 35DAP. Error bars represent the means ± SD, and the asterisks represent significant differences from the CK, with *p < 0.05 or ^**^p < 0.01. CK, the control; TR, shading treatment.

As described above, total GSLs concentration in siliques was significantly higher in shading treatment of both GH06 and ZY821 than that in the control (Figure 2). Similarly, 12 common differential metabolites in GH06 and ZY821 belonged to GSL class of known compounds (Figure 3C and Supplementary Table 3). Therefore, 10 significantly different GSLs constituents at 35 DAPS were selected for further study (Figure 4). In total, 4 aliphatic GSLs, i.e., glucoerucin, glucoberteroin, glucoalyssin (RT = 1.28 min), and glucoalyssin (RT = 1.70 min) were increased in shaded siliques (Figures 4A–D). Meanwhile, 4 indolic GSLs, (tryptophan, glucobrassicin, 4-hydroxyglucobrassicin, and 4-methoxyglucobrassicin) (Figures 4E–H) and 2 aromatic GSLs (glucotropaelin and gluconasturtiin) (Figures 4I,J) showed the similar accumulation patterns, which obviously increased in siliques under shading treatment at 35DAP (Figures 4A–J), indicating that the contents of GSLs had higher accumulation levels in siliques under shading than that in the control. Correspondingly, we found that the accumulation levels of GSLs had significantly decreased in seeds of shaded siliques (Figure 4K). Hence, we speculated that the GSLs transportation might be inhibited from siliques to seeds after shading treatment.

**FIGURE 5:**
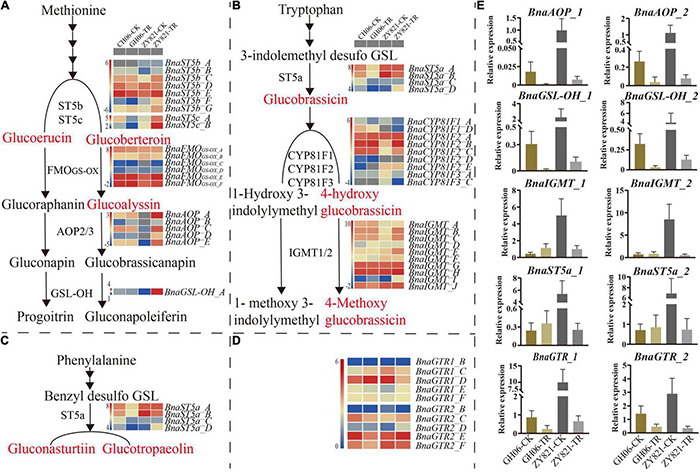
The relative expression levels of GSLs genes in siliques of rapeseed at 35DAP **(A)** The gene expression profiles of Aliphatic GSL pathway at 35DAP; **(B)** The gene expression profiles of Indolic GSL pathway at 35DAP; **(C)** The gene expression profiles of Aromatic GSL pathway at 35DAP; **(D)** The gene expression profiles of GSL transporters (*BnaGTR1* and *BnaGTR2*) at 35DAP; **(E)** RT-qPCR validation of GSLs (biosynthetic and transporter) gene expression from the RNA-seq data at 35DAP. Red highlighted compounds are significantly differential common GSLs in GH06 and ZY821. Error bars represent the means ± SD of biological replicates. CK, the control; TR, shading treatment; DAP, days after pollination.

To test the hypothesis that light induces the biosynthesis and transport of GSLs from siliques to seeds, expression profiles of *GSLs* genes were investigated in siliques at 35DAP under control and shading treatment using RNA-seq analysis (Figures 5A–D). The expression profiles of most of the genes were high under the normal condition as compared to shading. These RNA-seq results were further validated by RT-qPCR analysis. Hence, *AOP2* and *GSL-OH* from aliphatic GSLs, *IGMT* from indolic GSLs and *ST5a* from aromatic GSLs were selected in this study, respectively (Supplementary Tables 4, 5). The *GSL-OH* and *AOP2* showed significantly higher expression levels under normal than shading treatment in both GH06 and ZY821 (Figure 5E). However, the expression patterns of *IGMT1/2* and *ST5a* in siliques of GH06 were slightly higher under shading treatment than in the control, which were significantly suppressed in siliques of ZY821 after shading, indicating that these might be involved in the background of rapeseed (Figure 5E). Importantly, the expression levels of transporter genes, *BnGTR1* and *BnGTR2*, were almost significantly decreased in siliques of GH06 and ZY821 after shading treatment (Figure 5E), suggesting that lights are associated with the GSLs transportation in siliques of rapeseed. In summary, light may be an essential factor for the accumulation and transportation of GSLs in siliques of rapeseed.

### Analysis of *Cis*-Regulatory Elements in the Promoter Regions of *BnaGTR* Genes

To well understand the possible roles of *BnaGTRs* in response to light, sequences of 1.5 kbp upstream of the initiation codon ATG of 12 *BnaGTR* members were obtained from rapeseed genomic DNA and subjected for analysis on the Plant CARE database. Results showed that various *cis*-acting elements related to light stress, including AE-box, Box 4, chs-Unit 1 m1, GA-motif, GT1-motif, I-box, and TCT-motif (Supplementary Figure 3 and Supplementary Table 6). Furthermore, the motifs (GA-motif, GT1-motif, Box-4, and I-box) that are the light-responsive elements are highly conserved and widely present in the *BnaGTRs* promoters, indicating that the functions of *BnaGTRs* might be associated with the light. Our results provide the information for elucidating the mechanism of *BnaGTRs* in rapeseed.

## Discussion

Light is an important factor that affects plant development, morphogenesis, growth, and secondary metabolite synthesis (Fukuda et al., 2002; Nishimura et al., 2007). Previous studies showed that photosynthesis in the silique walls is the main source for the seed filling (Hua et al., 2012; Tan et al., 2015). As we expected, the phenotypes of siliques and seeds lost the green color after masking with aluminum foil at 35 DAP (shading for 20 days) (Figure 1), showing premature development of siliques and seeds showed premature senescence, color differences and size reduction, and the shapes of mature seeds in shading were wrinkled as compared to the round seeds of controlled conditions (Figure 1), which were also reported by Tan et al. (2015) to analyze the effects of light on dynamic metabolites of developing seeds and siliques of *B. napus*.

Numerous metabolites, such as phenolic acid, flavonoids, GSLs, and other hydroxycinamic acid are widely detected in rapeseed (Qu et al., 2013, 2020; Yin et al., 2019; Shen et al., 2021), which were easily affected by different kinds of environmental factors in *B. napus* (Naguib et al., 2012). However, siliques are the dominant photosynthetic organ for supporting the seed filling during late stages in rapeseed (Tan et al., 2020), which should be acted as one of the main parameters for secondary metabolite synthesis. In this study, we identified 249 differential metabolites from siliques of rapeseed through UPLC–HESI–MS/MS, which were classified into five major classes and unknown compounds, including phenolic acids, flavonoids, GSLs, lipids, and other hydroxycinamic acids (Supplementary Table 1). Furthermore, most of these compounds had been also identified from the seeds of *B. napus*, such as 29 phenolic acids, 38 flavonoids, and 22 GSLs from siliques and seeds (Shao et al., 2014; Heras et al., 2016; Li et al., 2017). These findings suggest that these compounds were distributed extensively in siliques and seeds of *B. napus*.

Glucosinolates are sulfur-rich secondary metabolites that are extensively distributed in *Brassicaceae* family (Bell et al., 2018; Park et al., 2019). They are not only important regulators for plant defense against pests and pathogens, but also control the oil quality (Ishida et al., 2014). Meanwhile, leaves and silique walls are the main production sites for the synthesis of GSLs, but seeds are their ultimate storage sites (Chen et al., 2001; Nour and Halkier, 2008; Yu et al., 2014). As the dominant photosynthetic organ, however, results showed that silique walls play crucial roles in supporting the seed filling during late stages (Tan et al., 2020). In this study, we also found that metabolites showed obvious variations after shading the siliques (Figure 2 and Supplementary Table 2), suggesting that light is essential for metabolic activity in the siliques. Previous results showed that GSLs in seeds were mainly originated from the siliques in rapeseed (Du and Halkier, 1998; Jørgensen et al., 2015). However, they had widely been reported in *Brassica* vegetables, such as *Brassica rapa* (Padilla et al., 2007; Barbieri et al., 2008; Kim et al., 2010; Baek et al., 2016; Klopsch et al., 2018), *Brassica oleracea* (Rungapamestry et al., 2006; Wang et al., 2012), *Brassica campestris* (Chen et al., 2008), *B*. *napus* (Kittipol et al., 2019), and *Brassica juncea* (Sun et al., 2019). In addition, numerous metabolites had also been identified from seeds of *Brassica* species (Shao et al., 2014; Wang et al., 2018; Qu et al., 2020; Shen et al., 2021), but few were reported in siliques of *B*. *napus*. Herein, we identified 22 GSLs from rapeseed siliques (Supplementary Table 2), of which 10 common differential GSLs between GH06 and ZY821 showed significant differences after shading the siliques (Figure 4 and Supplementary Table 3), indicating that these GSLs were synthesized in the siliques of rapeseed, and affected by light factor.

Recently, suitable lights are effective in increasing the accumulation of GSLs and phenolics in *B*. *napus* sprouts (Park et al., 2019, 2020). However, how light induces GSLs synthesis and transport in silique walls and seeds remains unclear. Previous results showed that GSLs follow the circadian rhythms by varying continuously in a day (Rosa and Rodrigues, 1998), which were decreased in *Brassica oleracea* at day time, but their accumulation started to increase at night (Rosa et al., 1994). In addition, the amount of GSLs showed different fluctuations with light quality and intensity (Pérez et al., 2008). In this study, total GSLs contents had obviously accumulated in siliques under shading (Figures 2, 4A–J), in accordance with previous results (Rosa et al., 1994). Meanwhile, their amounts decreased in the seeds (Figure 4K), suggesting that the transportation of GSLs should be inhibited from siliques to seeds in rapeseed.

**FIGURE 6:**
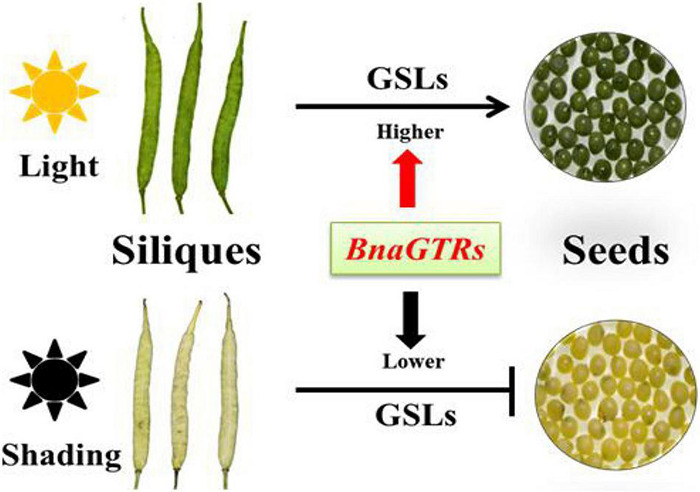
Hypothetical regulatory network of GSLs accumulation and transportation in siliques and seeds of *Brassica napus* (*B. napus)*.

In *Arabidopsis*, the GSLs biosynthetic pathway is widely known as “model” for secondary metabolites, which has also been studied extensively (Halkier and Gershenzon, 2006; Sønderby et al., 2010; Lu et al., 2018; Chhajed et al., 2020; Nguyen et al., 2020). Therefore, we also investigated the expression profiles of some GSLs key genes involved in the biosynthetic pathway of GSLs (Figure 5), results showed that they were highly consistent with the corresponding metabolites (Figure 4), which confirmed that light factors play crucial roles in the biosynthesis of GLSs in siliques of rapeseed. Among them, *GTR1* and *GTR2* genes are reported as the transporter genes for loading GSLs that are transported after their maturity from different parts of *B. napus* to the seeds through *GTRs* (transporter genes) (Nour and Halkier, 2008; Jørgensen et al., 2015). In this study, we have identified 12 homologous genes of *GTR1* and *GTR2* in *B. napus* (Supplementary Table 4). In *A. thaliana*, *GTR1* and *GTR2* belong to nitrite, and peptide transporter family are specific transporter proteins for aliphatic and indolic GSL (Nour et al., 2012). In addition, loss of function mutation of *GTR* genes in *B. rapa* and *B. juncea* resulted in a 60–70% reduction in GSL accumulation in seeds (Nour et al., 2017). In *B. juncea*, the knockdown of *GTR1* had the least effects on the transport of GSL from siliques and leaves to seeds but *GTR2* knockdown resulted in lower accumulation in seeds but a higher amount of GSL in siliques and leaves (Nambiar et al., 2021). In Chinese kale, three GTR1 genes have been cloned as *BocGTR1a*, *BocGTR1b*, and *BocGTR1c*. Among these, *GTR1a* and *GTR1c* were highly expressed in different tissues like leaves and buds, and their silencing caused a reduction in GSL accumulation in seeds (Jiang et al., 2019). Our findings also showed that all the identified *BnaGTRs* showed different expression patterns. As we expected, however, the expression levels of *BnaGTRs* had significantly decreased after shading treatment (Figure 5E). Meanwhile, the presence of *cis*-regulatory elements in the 5′ flanking site of promoter regions of these 12 *BnaGTR* genes, including AE-box, Box 4, chs-Unit 1 m1, GA-motif, GT1-motif, I-box, and TCT-motif (Supplementary Figure 3 and Supplementary Table 6), are listed as the elements for light responsiveness in *B. napus* (Kumar et al., 2009; Lohani et al., 2021; Shahmir and Pauls, 2021; Zhu et al., 2021). Hence, we proposed that transporter genes are most likely to be affected by shading, which led to a very low expression of *BnaGTR* genes, and caused an interruption in the transportation of GSLs from siliques to seeds in the treatment which ultimately resulted in their high accumulation in siliques (Figure 6). This depicted that shading not only affected the expression of biosynthetic genes but also downregulated the expression of GTR genes of GSLs. Our results provide the new insights into well understanding the metabolites of siliques, as well as clues for elucidating the mechanisms of GSLs accumulation and transportation in rapeseed.

## Data Availability Statement

The original contributions presented in the study are publicly available. This data can be found here: National Center for Biotechnology Information (NCBI) BioProject database under accession number PRJNA770894.

## Author Contributions

KL and CQ: conceptualization, resources, writing—review and editing, supervision, and project administration. FK, SS, and NY: methodology. FK, SS, and RH: software. QZ, YM, and JL: validation. XX: formal analysis. YJ: investigation. FK, SS, and CQ: writing—original draft preparation. CQ: visualization. JL, KL, and CQ: funding acquisition. All authors have read and agreed to the published version of the manuscript.

## Conflict of Interest

The authors declare that the research was conducted in the absence of any commercial or financial relationships that could be construed as a potential conflict of interest.

## Publisher’s Note

All claims expressed in this article are solely those of the authors and do not necessarily represent those of their affiliated organizations, or those of the publisher, the editors and the reviewers. Any product that may be evaluated in this article, or claim that may be made by its manufacturer, is not guaranteed or endorsed by the publisher.

## Footnotes

1
http://www.reifycs.com/AbfConverter/index.html

2
http://prime.psc.riken.jp/compms/msdial/main.html#MSP

3
www.metaboanalyst.ca

## Abbreviations

GSL: glucosinolate
AOP: 2-oxoglutarate-dependent dioxygenase
GSL-OH: glucosinolate hydroxylase
IGMT: indole glucosinolate O-methyltransferase
ST5a: sulfotransferase 5a
Bna: *Brassica napus*
GTRs: glucosinolate transporter genes
DAP: Days after pollination
HPLC: High Performance Liquid Chromatography
HESI: heated electrospray ionization
MS/MS: tandem mass spectrometry
RT: retention time
SD: standard deviation
UPLC: ultrahigh-performance liquid chromatography
FPKM: fragments per kilobase of exon model per million
DEGs: difffferentially expressed genes
FC: fold changes
PCA: principal component analysis
MOF: Ministry of Finance of the People’s Republic of China
MARA: Ministry of Agriculture and Rural Affairs of the People’s Republic of China.
