# Supplementary material for: Metabolite Characteristics Analysis of Siliques and Effects of Lights on the Accumulation of Glucosinolates in Siliques of Rapeseed
Source: Front Plant Sci. 2022 Feb 16;13:817419. doi: 10.3389/fpls.2022.817419 (PMC8888874; doi:10.3389/fpls.2022.817419)
Supplement: Supplementary file 7 [file Data_Sheet_1.PDF]

Supplementary Figures:

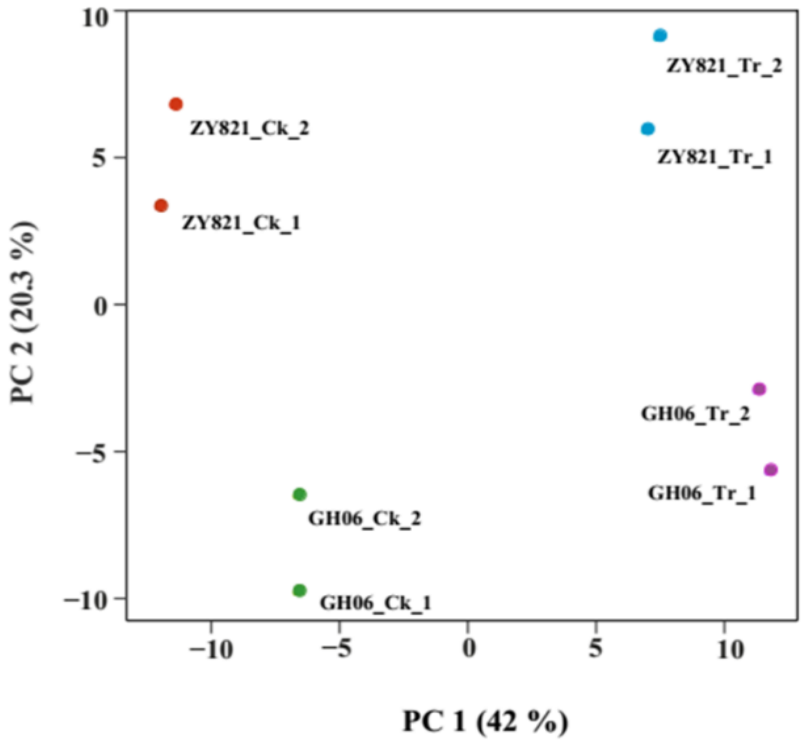

Figure S1: Principal component analysis (PCA) of metabolites in siliques of rapeseed. Different colored dots represent the GH06 and ZY821. CK, the control; TR, shading treatment.

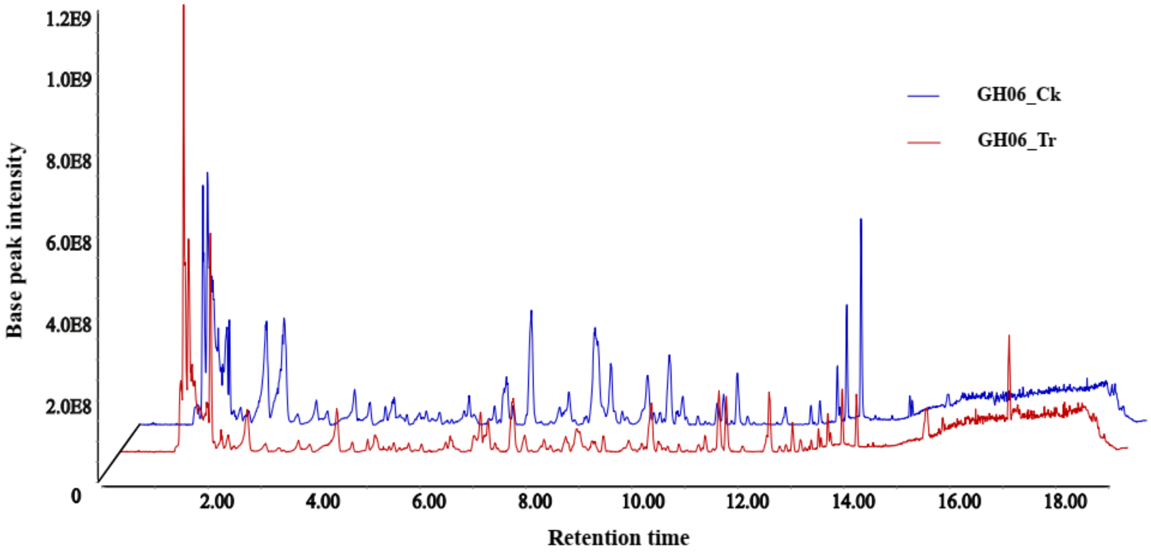

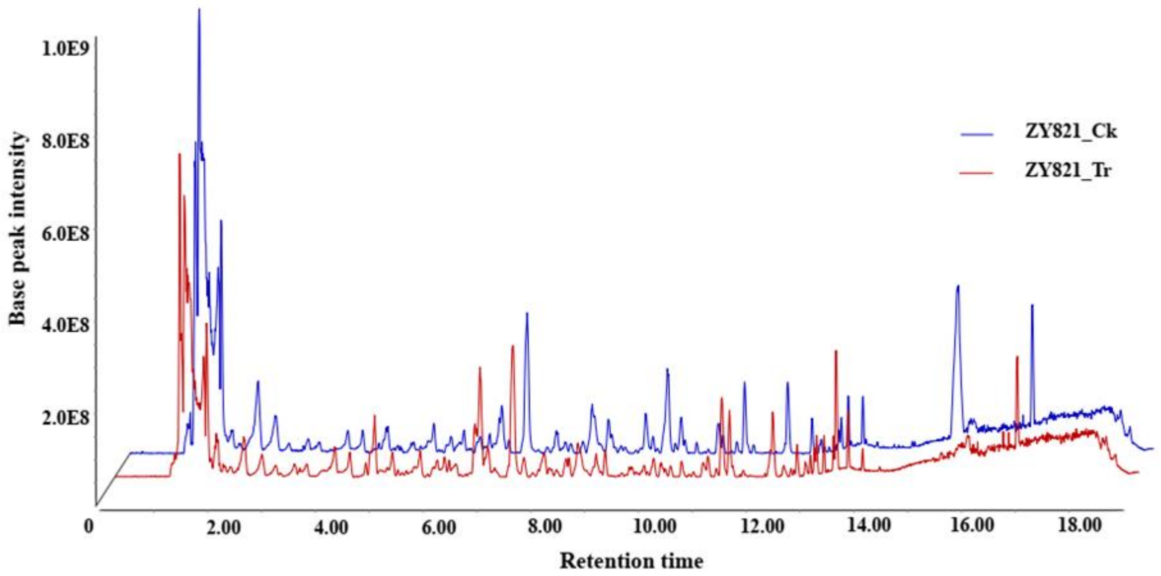

Figure S2: Total Ion Chromatogram (TIC) of siliques of rapeseed varieties (GH06 and ZY821) under Full MS-ddMS^2 (m/z =100–1500) at 35DAP. The blue and red lines indicate control (Ck) and shading (Tr); DAP; Days after pollination.

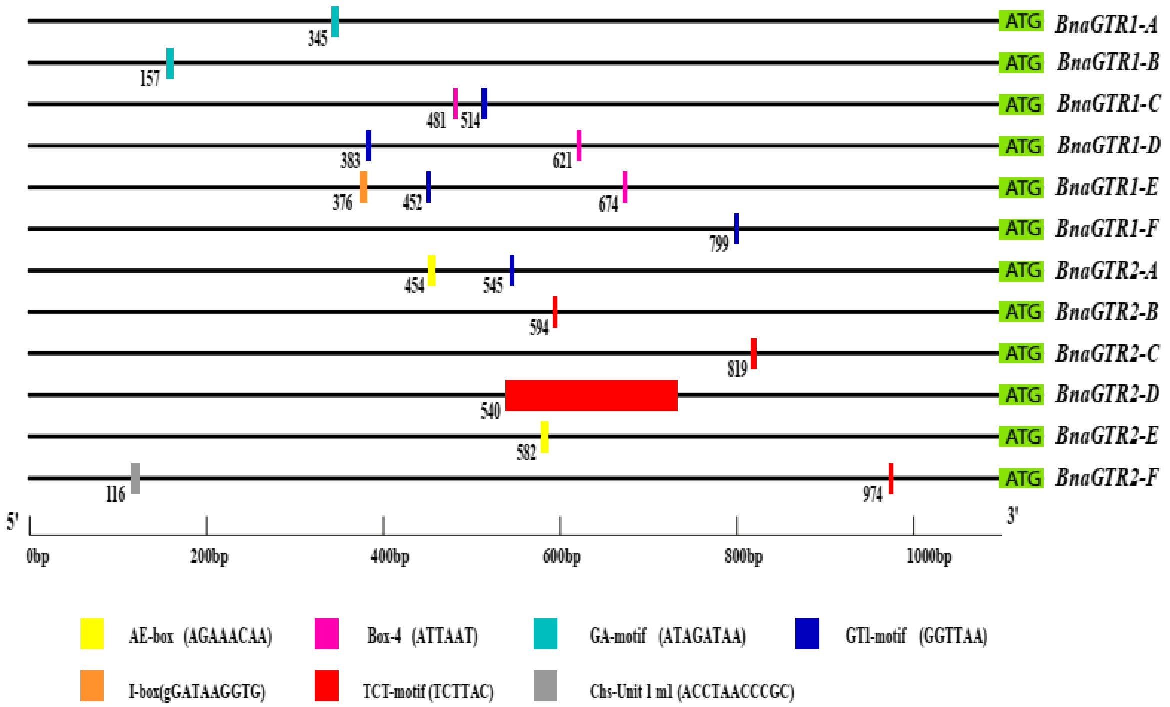

Figure S3. Analysis of *Cis* regulatory elements from 1500 bp nucleotides in upstream regions of *BnaGTR*s for light responsiveness.
